# Supplementary material for: t6A and ms2t6A Modified Nucleosides in Serum and Urine as Strong Candidate Biomarkers of COVID-19 Infection and Severity
Source: Biomolecules. 2022 Sep 3;12(9):1233. doi: 10.3390/biom12091233 (PMC9496545; doi:10.3390/biom12091233)
Supplement: Supplementary file 1 [file biomolecules-12-01233-s001.zip › biomolecules-1880702-supplementary.pdf]

SUPPLEMENTARY DATA

Figure S1. Correlations between modified nucleosides in serum and clinical indicators of COVID-19.

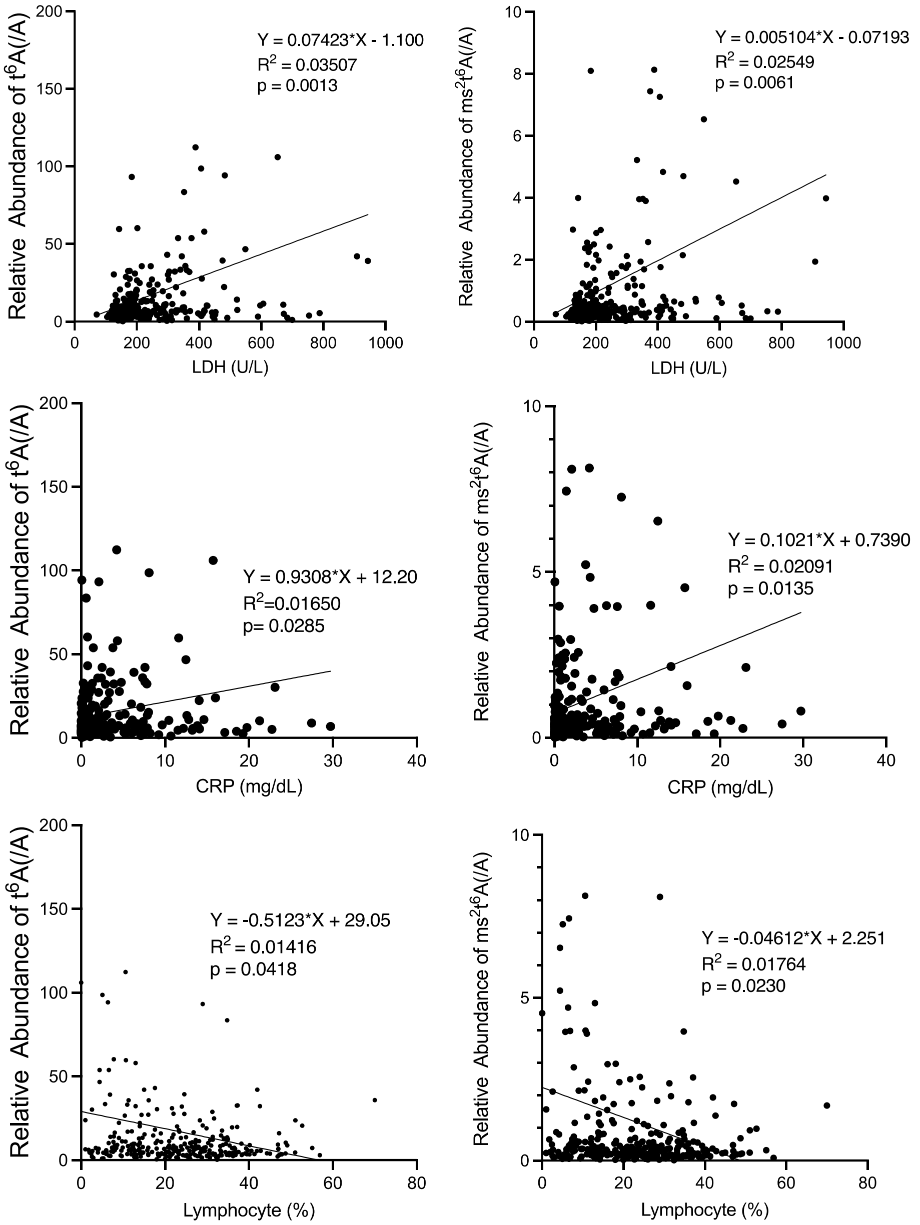

Abbreviations: WBC: white blood cells; LDH: lactate dehydrogenase; CRP: C-reactive protein

Table S1. MRM transition parameters for modified nucleosides.

Nucleosides	Precursor ion (m/z)	Product ion (m/z)
Ψ	245.10	209.00
Cm	258.25	112.05
m^1A, m^6A	282.30	150.05
Um	259.00	113.00
Gm	298.10	152.10
Im	283.10	137.10
m^2_2G	312.10	180.10
t^6A	413.10	281.10
Am	282.10	136.00
ms^2t^6A	459.10	327.10

$m^6{}_2A$	296.00	164.00
m^6t^6A	427.30	136.00
m^6Am	296.00	150.05
D	247.00	115.00
U	245.00	113.00
G	284.00	152.05
C	244.20	112.10
A	268.00	136.00

Ψ: pseudouridine; Cm: 2'-*O*-methylcytidine; m^1A: N^1-methyladenosine; m^6A: N^6-methyladenosine; Um: 2'-*O*-methyluridine; Gm: 2'-*O*-methylguanosine; Im: 2'-*O*-methylinosine; $m^2{}_2G$: N^2,N^2-dimethylguanosine; t^6A: N^6-threonylcarbamoyladenosine; Am: 2'-*O*-methyladenosine; ms^2t^6A: 2-methylthio-N^6-threonylcarbamoyladenosine; $m^6{}_2A$: N^6,N^6-dimethyladenosine; m^6t^6A: N^6-methyl-threonylcarbamoyladenosine; m^6Am: N^6,2'-*O*-dimethyladenosine; D: dihydrouridine; U: uridine; C: cytidine; G: guanosine; A: adenosine.

Table S2. Information of the patients with other infectious diseases.

Bacterial infection

Patient number	Diagnosis	Physical examinations or tests related diagnosis
Patient 1	Bacterial pneumoniae and pleurisy	Fever, elevated CRP, pneumoniae image on CT scan, and bacterial detection in turbid pleural fluid.
Patient 2	Bacterial pneumoniae	Fever, elevated CRP, pneumoniae image on CT scan, and bacterial detection by Gram staining of sputum.
Patient 3	Bacterial pneumoniae	Fever, elevated CRP, pneumoniae image on CT scan, and bacterial detection by Gram staining of sputum.
Patient 4	Bacterial pneumoniae and pleurisy	Fever, elevated CRP, pneumoniae image on CT scan, and bacterial detection in turbid pleural fluid.
Patient 5	Bacterial pneumoniae	Fever, elevated CRP, pneumoniae image on CT scan, and bacterial detection by Gram staining of sputum.
Patient 6	Bacterial pneumoniae and pleurisy	Fever, elevated CRP, pneumoniae image on CT scan, and bacterial detection in turbid pleural fluid.
Patient 7	Bacterial pneumoniae	Fever, elevated CRP, pneumoniae image on CT scan, and bacterial detection by Gram staining of sputum.
Patient 8	Bacterial pneumoniae and pleurisy by *Pseudomonas aeruginosa*	Fever, elevated CRP, pneumoniae image on CT scan, and bacterial detection in blood culture test.
Patient 9	Bacterial pneumoniae	Fever, elevated CRP, pneumoniae image on CT scan, and bacterial detection by Gram staining of sputum.
Patient 10	Bacterial pneumoniae by *Streptococcus pneumoniae*	Fever, elevated CRP, pneumoniae image on CT scan, and rapid urinary antigen test of *Streptococcus pneumoniae* was positive.

Patient 11	Bacterial pneumoniae	Fever, elevated CRP, pneumoniae image on CT scan, and bacterial detection by Gram staining of sputum.
Patient 12	Bacterial pneumoniae	Fever, elevated CRP, pneumoniae image on CT scan, and bacterial detection by Gram staining of sputum.
Patient 13	Bacterial pneumoniae by *Streptococcus pneumoniae*	Fever, elevated CRP, pneumoniae image on CT scan, and rapid urinary antigen test of *Streptococcus pneumoniae* was positive.
Patient 14	Bacterial pneumoniae	Fever, elevated CRP, pneumoniae image on CT scan, and bacterial detection by Gram staining of sputum.
Patient 15	Bacterial pneumoniae	Fever, elevated CRP, pneumoniae image on CT scan, and bacterial detection by Gram staining of sputum.
Patient 16	Bacterial pneumoniae by *Haemophilus influenzae*	Fever, elevated CRP, pneumoniae image on CT scan, and bacterial detection by blood culture test.
Patient 17	Bacterial pneumoniae	Fever, cough, elevated CRP, pneumoniae image on CT scan, and bacterial detection by Gram staining of sputum.
Patient 18	Bacterial pneumoniae	Fever, cough, elevated CRP, pneumoniae image on CT scan, and bacterial detection by Gram staining of sputum.

Viral infection

Patient number	Diagnosis	Physical examinations or tests related diagnosis
Patient 1	Influenza A	Fever and the results of Rapid antigen test was positive.
Patient 2	Influenza B	Fever and the results of Rapid antigen test was positive.
Patient 3	Influenza A	Fever and the results of Rapid antigen test was positive.
Patient 4	Influenza A	Fever and close contact with Influenza A patient.
Patient 5	Influenza A	Fever and the results of Rapid antigen test was positive.
Patient 6	Influenza B	Fever and the results of Rapid antigen test was positive.
Patient 7	Influenza B	Fever and the results of Rapid antigen test was positive.
Patient 8	Influenza A	Fever and the results of Rapid antigen test was positive.
Patient 9	Influenza A	Fever and close contact with Influenza A patient.
Patient 10	Influenza A	Fever and the results of Rapid antigen test was positive.
Patient 11	Upper respiratory tract infection	Fever and sore throat.
Patient 12	Influenza A	Fever and the results of Rapid antigen test was positive.
Patient 13	Influenza A	Fever and the results of Rapid antigen test was positive.
Patient 14	Influenza A	Fever and the results of Rapid antigen test was positive.
Patient 15	Influenza A	Fever and the results of Rapid antigen test was positive.
Patient 16	Influenza A	Fever and the results of Rapid antigen test was positive.
Patient 17	Influenza A	Fever and the results of Rapid antigen test was positive.
Patient 18	Influenza A	Fever and the results of Rapid antigen test was positive.
Patient 19	Influenza A	Fever and the results of Rapid antigen test was positive.

Patient 20	Influenza A	Fever and the results of Rapid antigen test was positive.
Patient 21	Influenza A	Fever and the results of Rapid antigen test was positive.
Patient 22	Influenza A	Fever and the results of Rapid antigen test was positive.
Patient 23	Influenza A	Fever and the results of Rapid antigen test was positive.
Patient 24	Influenza A	Fever and the results of Rapid antigen test was positive.
